# Diagnosing Pseudotumor Cerebri: An Age-based Approach

**DOI:** 10.15844/pedneurbriefs-34-19

**Published:** 2020-12-18

**Authors:** Tuhina Govil-Dalela, Lalitha Sivaswamy

**Affiliations:** 1 Department of Child Neurology, Children’s Hospital of Michigan, Wayne State University, Detroit, MI

**Keywords:** Idiopathic Intracranial Hypertension, Pseudotumor Cerebri, Pediatrics, Headache, Papilledema

## Abstract

Investigators from Hillel-Yaffe, Carmel, and Bnai Zion Medical Centers in Israel studied the comparative clinical presentations and predisposing factors for idiopathic intracranial hypertension (IIH) across age groups

Investigators from Hillel-Yaffe, Carmel, and Bnai Zion Medical Centers in Israel studied the comparative clinical presentations and predisposing factors for idiopathic intracranial hypertension (IIH) across age groups. They retrospectively evaluated 72 patients and compared their data using pooled analyses with 1499 patients from previously published literature. Modified Dandy criteria were used for the diagnosis of IIH. They found that female predominance and association of obesity with IIH increases with age. As expected, headache was the most common symptom across age groups. Vomiting was seen in 10% of children, 45% of adolescents, and 8% of adults (P<0.01), while dizziness was reported only in adolescents (9%) and adults (10%). The incidence of papilledema and CSF opening pressure was not significantly different across age groups. Comparison of this data set with the pooled literature analysis showed only minor differences in numbers. [1]

COMMENTARY. The diagnostic criteria for IIH have evolved. The modified Dandy criteria put forth in 1985 used symptoms, signs, elevated opening pressure, and CT findings for diagnosis. The Pseudotumor Cerebri Syndrome (PTCS) criteria, developed in 2013, removed the components emphasizing subjectivity (symptoms) and gave specific CSF opening pressure parameters for children with IIH [2]. Although a common undercurrent amongst the pediatric population is an inability to clearly state their symptoms, the complete exclusion of symptoms and reliance on clinical signs and testing alone may lead to underdiagnosis, problematic for a disorder that may lead to irreversible vision loss [3]. From a practical standpoint, symptoms form one of the data points for follow-up care of children and determine response to treatment. The absence of headache in the diagnostic criteria presents a practical conundrum because how else would the clinician’s attention be drawn towards eliciting signs of IIH? Further, as noted by Aylward et al., even papilledema -long considered an essential component for the diagnosis -may not be present in a subset of children with IIH [4].

Since the 1990s and until as recently as 2014, most of the published literature on IIH focused on and explicitly stated that IIH was a disease of young, obese women and was almost exclusively seen in this patient population [5,6]. Although some studies specifically geared towards IIH in children, they primarily dealt with risk factors and did not differentiate clinical presentations or compare different age groups [7].

In summary, this study provides practicing pediatric neurologists a categorization of various clinical presentations of IIH in different age groups, so the diagnosis is hopefully not missed in children not belonging to the “typical” patient population. It also provides a framework for further prospective studies, identifies data points for creating more inclusive diagnostic testing with high sensitivity (though it may be at the cost of specificity) for early diagnosis of IIH, and hopefully lays the groundwork for creating data-driven treatment pathways. The former remains a striking void in the field of pediatric IIH – one that such large-scale analyses can potentially fill in the not-too-distant future.

## Disclosures

The authors have declared that no competing interests exist.
